# Use of Adsorption Properties of Resin for Water Sample Preparation in Voltammetric Determination of Se(IV) Using Bismuth Microelectrode

**DOI:** 10.3390/molecules29235501

**Published:** 2024-11-21

**Authors:** Malgorzata Grabarczyk, Marzena Fialek, Cecylia Wardak

**Affiliations:** Department of Analytical Chemistry, Institute of Chemical Sciences, Faculty of Chemistry, Maria Curie-Sklodowska University, 20-031 Lublin, Poland; marzena.fialek@mail.umcs.pl (M.F.); cecylia.wardak@mail.umc.pl (C.W.)

**Keywords:** selenium, resin, voltammetry, natural waters

## Abstract

This paper proposes a simple, environmentally friendly, and efficient procedure for preparing natural water samples for the voltammetric determination of trace amounts of Se(IV). The method is based on premixing a sample with Amberlite XAD-7 resin at 50 °C. The composition of the 10 mL solution consists of the sample to be analysed, 0.1 mol L^−1^ of acetate buffer at pH = 4.0, and 0.5 g of Amberlite XAD-7. After 2 min of stirring, a voltammetric measurement is carried out using a fixed bismuth microelectrode using the following potentials: −2.5 V for 2 s and −0.55 V for 30 s. The voltammetry is recorded by varying the potential from −400 mV to −1000 mV. An undisturbed Se(IV) signal is obtained in the presence of 10 mg L^−1^ of Triton X-100, 5 mg L^−1^ of SDS, 10 mg L^−1^ of CTAB, 5 mg L^−1^ of Rhamnolipid, 5 mg L^−1^ of HA, 10 mg L^−1^ of FA, and 2 mg L^−1^ of NOM. The validity of the developed procedure is checked by analysing the certified reference materials SPS-SW1 (surface water) and TM-25.5 (Lake Ontario water) additionally enriched with surfactants and humic substances.

## 1. Introduction

Continuous developments in analytical chemistry enable new insights into the presence and roles of many elements present in the environment. One such element is selenium, which occurs in the environment in different forms with drastically differing properties and, consequently, toxicities. For this reason, the determination of selenium is not simply a matter of ascertaining its presence and quantifying its total content in the sample analysed. In this case, it is necessary to use analytical methods that allow for speciation, i.e., the determination of only a selected form of selenium. It is important that other forms of selenium potentially present in the sample analysed do not affect the signal of the form being determined. Such methods unquestionably include stripping voltammetry, which, in many cases, allows for direct speciation analysis without the necessity of masking or removing other forms of the determined element, which accompany the determined form, from the analysed sample. This is the case for the voltammetric speciation analysis of selenium, which allows for direct determinations of Se(IV) in aqueous samples in the presence of Se(VI). In natural waters, selenium occurs mainly in the form of the inorganic compounds Se(IV) and Se(VI), of which Se(IV) is the most toxic and best absorbed by the human body [1,2,3,4,5,6]. Selenium is a natural component of the Earth’s crust. A greater accumulation of it is observed in sulphur deposits. Selenium contained in volcanic rocks is carried by weathering processes into the atmosphere and, from there, into the waters of oceans, seas, and lakes, and it is also partly taken up by plants. Directly from weathering rocks, selenium enters flowing and groundwater and, as a result of hypergenic (epidiagenetic) and biogeochemical processes, also enters the soil. From the soil environment, selenium is taken up by plants, and through them, together with plant nutrients, enters the human body. Thus, the amount of selenium taken up by humans is supplemented by the ingestion of aquatic flora and fauna and by inhalation from the ambient air. Together with dead plants and excretions from humans and animals, part of the selenium taken up is returned to the soil. Another part, however, is transferred by plants into the atmospheric air in the form of volatile compounds. In addition to naturally occurring selenium, there is also additional selenium pollution in the environment. Environmental pollution by selenium is mainly related to the combustion processes of coal and petroleum products and the mining and processing of lead and copper ores. Anthropogenic sources of pollution also include the pharmaceutical industry, the fat industry, and the production of dyes. Nitrogen and phosphorus mineral fertilizers may also contribute to soil contamination with this element [6,7,8,9,10,11].

Controlling the amount of selenium in the human environment has been of interest to researchers in many disciplines in recent years. It is a classic example of a geochemical problem with health aspects. From the point of view of health protection, geochemical problems are considered both in terms of environmental deficiencies of elements of fundamental importance for the human organism and excesses of harmful elements at their higher concentrations. Selenium belongs to this group of elements, which should only be considered useful in a narrow range of concentrations, while in excess, has a harmful effect. In drinking water, selenium concentrations generally do not exceed 10 µg/L. However, in selenium-rich areas, this concentration can be much higher. In seawater, the average selenium level is low and does not usually exceed 100 ng/L [12,13,14].

The vast majority of analytical methods dedicated to the speciation analysis of selenium require an additional step to separate the different forms of this element before measurement. Among these methods, we can primarily distinguish hydride generation techniques coupled with different detection systems, such as atomic absorption spectrometry (AAS), inductively coupled plasma atomic emission spectrometry (ICPAES), and inductively coupled plasma mass spectrometry (ICPMS) [15,16,17,18]. Also, high-performance liquid chromatography (HPLC) is now an established approach to perform the separation of selenium species [19,20,21,22], however, it requires coupled detector systems, which are poor in sensitivity or too expensive for most researchers to be equipped with. Other procedures based on a combination of preliminary separation methods with instrumental analysis, including graphite furnace atomic absorption spectrometric (GFAAS) determination coupled with coprecipitation or solid phase extraction, should also be mentioned [23,24].

Among the few methods that do not require a separation step is stripping voltammetry, which allows for the determination of one form of the element in the presence of the other. But in this method, it is also necessary to properly prepare the sample before measurement. This is mainly due to the fact that the stripping voltammetry method is very sensitive to the organic matrix present in environmental samples. One of the most common ways of removing this matrix is the mineralization process, but this is time-consuming and requires additional apparatus. In addition, speciation changes in the analysed element may occur during this process [25,26,27]. Another important aspect of voltammetric methods for green chemistry is the use of environmentally friendly working electrodes in the measurements. Therefore, the aim of our research was to develop a voltammetric procedure for the determination of selenium using a non-toxic electrode working simultaneously with simple and rapid sample preparation for analysis. For this purpose, the adsorption properties of the Amberlite XAD-7 resin were used. Nonpolar XAD resins are generally used for the adsorption of organic substances from aqueous systems and polar solvents. XAD-7 is the only “moderately polar” XAD resin now available. It has been used for the removal of organic pollutants form aqueous wastes, groundwater, and vapour streams [28,29]. This resin was previously used to eliminate interferences in the voltammetric determination of Se(IV), however, in that procedure, a toxic mercury electrode was used as the working electrode [30]. In the procedure proposed in this work, a solid bismuth microelectrode was proposed as the working electrode, which, in combination with the simple and rapid sample preparation using Amberlite XAD-7 resin, allowed for parameters competitive with those previously described in the literature.

## 2. Results and Discussion

### 2.1. Water Sample Preparation

It is well known that aqueous samples very often require pre-treatment before being analysed by voltammetry. This is primarily due to the interferences associated with this technique caused by the presence of surfactants and humic substances in aqueous environmental samples. These substances tend to block the surface of the working electrode, thus restricting the access of the depolarizer to its surface, resulting in the sensitivity deteriorating or, in some cases, the analytical signal disappearing altogether. This was also the case for the voltammetric analysis of Se(IV), where the influence of the organic matrix on the recorded analytical signal was very high [31,32].

A very effective yet simple and fast way to prepare a water sample prior to analysis is the use of Amberlite XAD-7 resin. This resin has been successfully employed in procedures for the determination of various elements using different electrodes [33,34,35]. It has also been used previously in a voltammetric procedure for the determination of Se(IV), where an environmentally toxic mercury electrode was used. In the procedure proposed in this work, an environmentally friendly bismuth microelectrode (BiµE) was used. In addition, the effect of temperature on the removal efficiency of interfering organics by the resin was also investigated.

The optimization of the following sample preparation parameters is described below: the mass of the resin, the mixing time with the resin, and the temperature of the mixing with the resin. Their appropriate combination will enable their rational, practical application and the appropriate selection of sample preparation conditions depending on the sample matrix. These experiments were carried out using Triton X-100 as a model substance, the effect of which on the signal mirrored that of the organic substances in the synthetic laboratory sample.

#### 2.1.1. Mass of Resin

**Figure 1:**
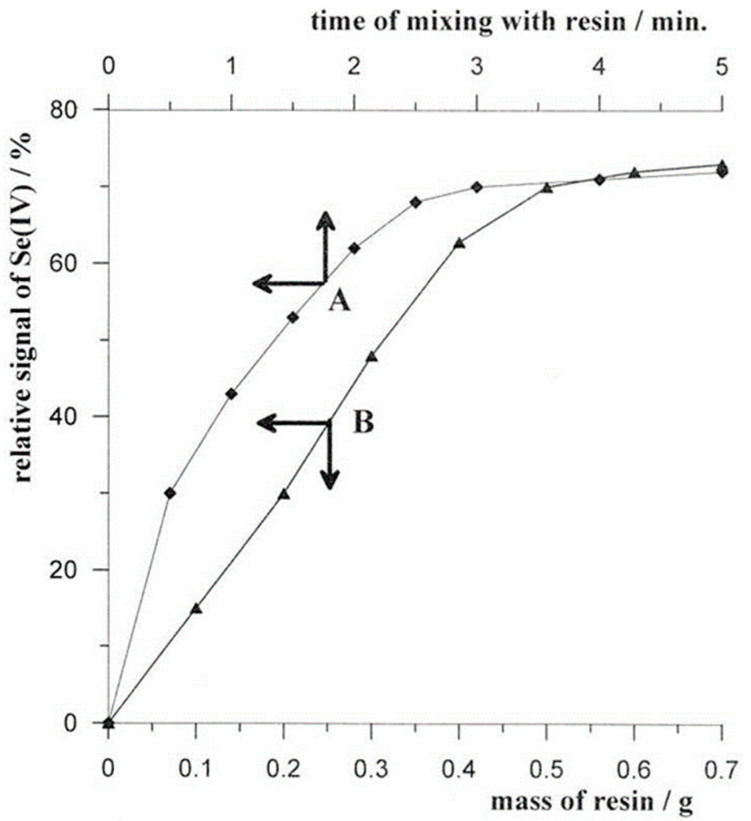
The influence of time of mixing with Amberlite XAD-7 resin (A) and mass of resin (B) on the relative voltamperometric signal of 3 × 10^−7^ mol L^−1^ Se(IV).

To investigate the effect of the resin weight on the efficiency of organic removal, the following solution of 10 mL was prepared: 0.1 mol L^−1^ of acetate buffer, 1 × 10^−7^ mol L^−1^ of Se(IV), and 15 mg L^−1^ of Triton X-100. Different masses of resin ranging from 0.05 to 0.7 mg were introduced into the solution and stirred for 2.5 min, followed immediately by a voltammetric measurement. The obtained results are presented in Figure 1B. It was found that, as the resin mass increased up to 0.5 mg, the selenium signal increased and then remained at a similar level. This indicates that, as the resin mass increased, more organic matter was removed by adsorption onto the resin, so the selenium signal increased as its attenuation was then lower. However, at resin weights above 0.5 g, the amount of organic matter removed no longer increased, so the selenium signal reached a constant value. Therefore, 0.5 g of resin was used as standard for further studies.

#### 2.1.2. Mixing Time with Resin

The precise amount of sample/resin mixing time required for effective organic removal was investigated. Most of the procedures described in the literature used 5 min for the mixing of the sample with the resin, and this was usually associated with the necessary 5 min deoxygenation of the sample before measurement. Since no deoxygenation was necessary in our proposed procedure for the determination of Se(IV) using BiµE, it was checked whether shorter mixing times with the resin could already be sufficient to obtain the desired results. For this purpose, solutions containing 0.1 mol L^−1^ of acetate buffer, 3 × 10^−7^ mol L^−1^ of Se(IV), 15 mg L^−1^ of Triton X-100, and 0.5 g of Amberlite XAD-7 resin were prepared. For each such solution, voltammetric measurements were taken after varying stirring times ranging from 0.5 to 5 min in 0.5 min increments. The obtained results are presented in Figure 1A. It was observed that, with an increasing mixing time from 0.5 to 2.5 min, the Se(IV) peak increased, and above this time, it remained constant. This confirms that, with an increasing stirring time, more organic matter was adsorbed onto the resin, so the selenium signal increased as it was less attenuated by organic matter. For times longer than 2.5 min, the amount of organic substations adsorbed on the resin no longer increased and, thus, the selenium signal no longer remained constant.

#### 2.1.3. Temperature of Mixing with Resin

**Figure 2:**
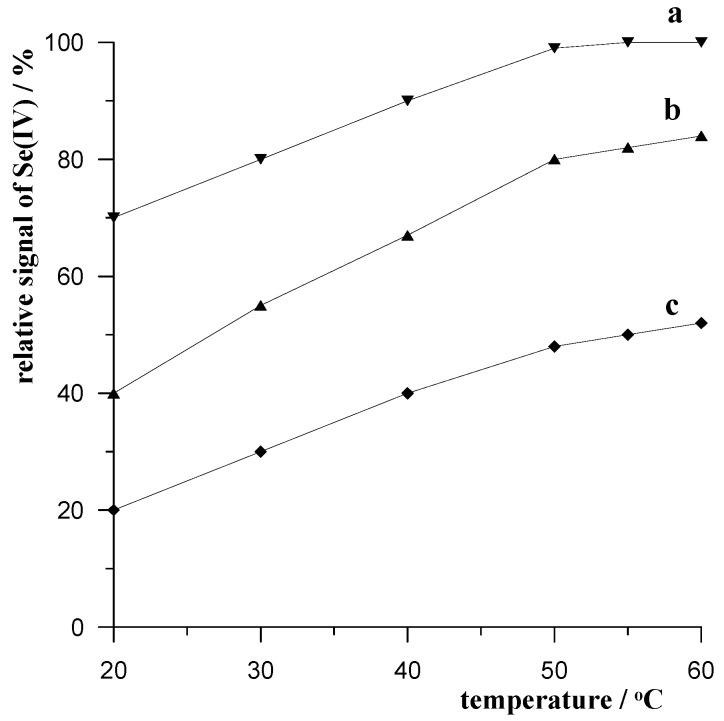
The influence of temperature on relative voltamperometric signal of 3 × 10^−7^ mol L^−1^ Se(IV) for different of Triton X-100 concentrations: 15 mg L^−1^ (a), 20 mg L^−1^ (b), and 25 mg L^−1^ (c).

As demonstrated in the work [36], an increase in temperature from 20 to 60 °C for mixing the surfactant-containing sample with the resin increased the removal efficiency of these substances by adsorption onto the resin. Therefore, in our proposed sample preparation procedure for voltammetric Se(IV) analysis, this parameter was also investigated. Experiments were conducted for a solution containing 0.1 mol L^−1^ of acetate buffer, 1 × 10^−7^ mol L^−1^ of Se(IV), 0.5 g of Amberlite XAD-7, and different concentrations of Triton X-100, 15, 20, and 25 mg L^−1^, for a resin mixing time of 2.5 min. The obtained results are presented in Figure 2. It was found that, with an initial temperature increase up to 50 °C, the selenium signal increased markedly, and above this temperature, the increase in magnitude was already negligible for all concentrations of Triton X-100 tested. This confirms previous observations that, with temperature, organic substances are more efficiently removed by the resin. In our procedure, measurements were carried out at room temperature as standard, but for samples rich in surfactants and humic substances, an increase in temperature to 50 °C is recommended for more efficient removal.

### 2.2. Organic Substances as Matrix of Aqueous Environmental Samples

A variety of organic compounds are present in surface waters. They include surfactants and humic substances. The surfactants in environmental waters are of anthropogenic origin, entering the environment as a result of human activities. This is primarily related to the development of a number of industries such as textiles, detergents and cosmetics, microelectronics, semiconductors, and now increasingly in medicine. With regard to chemical structure, we distinguish between non-ionic surfactants, anionic surfactants, cationic surfactants, and biosurfactants [37,38]. Humic substances are natural organic substances that are a mixture of aliphatic and aromatic substances. They are formed during the breakdown processes of plant and animal residues (humification). These residues are partially biodegradable. They can be synthesized by microorganisms as intermediates and undergo other chemical and physical transformations. Humic compounds can be classified as follows: fulvic acids—soluble in water in the pH range of 0–14 and yellow or yellow-brown in colour, humic acids—soluble in alkaline environments and brown or brown-black in colour, and humins—insoluble in water and black in colour. Humates account for approximately 50–75% of the dissolved organic carbon in environmental waters [39].

In our work, in order to test the broadest possible spectrum of organic substances present in surface waters, we used the following surfactants for laboratory testing: Triton X-100—non-ionic, sodium dodecyl sulphate (SDS)—anionic, cetyltrimethylammonium bromide (CTAB)—cationic, and Rhamnolipid—biosurfactant, and humic substances such as humic acid (HA), fulvic acid (FA), and natural organic material (NOM).

#### 2.2.1. Surface Active Substances

**Figure 3:**
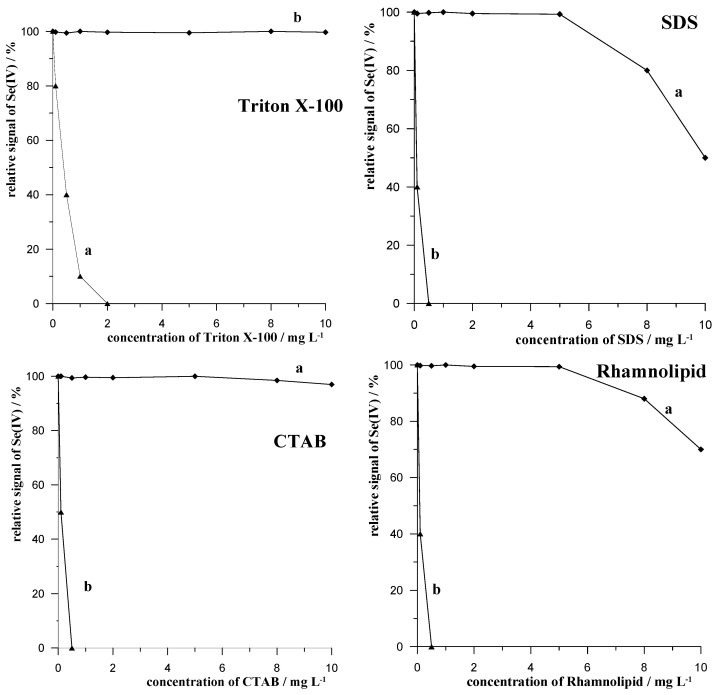
The influence of concentration of Triton X-100, SDS, CTAB, and Rahmnolipid on relative signal of Se(IV) in the absence (a) and presence (b) of the procedure of mixing with resin Amberlite XAD-7.

**Figure 4:**
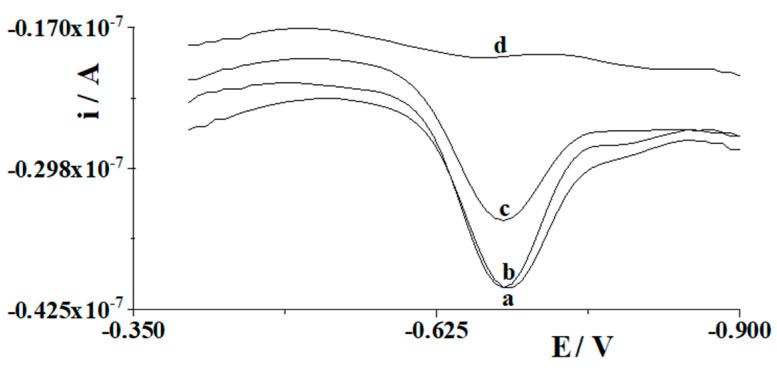
Voltammograms for 5 × 10^−8^ mol L^−1^ Se(IV) recorded in the presence of SDS: 0 (a), 0.5 mg L^−1^ (d), 5 mg L^−1^ (b), and 8 mg L^−1^ (c). Measurements were carried out without mixing with resin (a, d) and with mixing with 0.5 mg Amberlite XAD-7 resin for 2 min at 50 °C (b, c).

The effect on the analytical Se(IV) signal of the following surfactants, Triton X-100, SDS, CTAB, and Rhamnolipid, was precisely investigated. For this purpose, different concentrations of the above-mentioned surfactants in the concentration range from 0.1 to 20 mg L^−1^ were introduced into a 10 mL synthetic sample containing 0.1 mol L^−1^ of acetate buffer at pH = 4.0, 5 × 10^−8^ mol L^−1^ of Se(IV), and 0.5 g of Amberlite XAD-7. After 2 min of stirring in a thermostated vessel at 50 °C, voltammetric measurements were carried out as described in Chapter 2. The results obtained are presented in the graphs in Figure 3. For comparison, each graph also shows the Se(IV) signal recorded in the presence of the individual surfactants, but without the presence of the Amberlite XAD-7 resin. As can be seen, in each case, the presence of the resin made it possible to obtain an undisturbed Se(IV) signal, even in the presence of high surfactant concentrations. The acceptable non-decreased Se(IV) signal concentrations for Triton X-100, SDS, CTAB, and Rhamnolipid were 10, 5, 10, and 5 mg L^−1^, respectively, whereas in the absence of resin and the presence of 2 mg L^−1^ of Triton X-100 and 0.5 mg L^−1^ of SDS, CTAB, and Rhamnolipid, the Se(IV) signal was completely suppressed. Example voltammograms recorded for 5 × 10^−8^ mol L^−1^ of Se(IV) in the presence of different concentrations of SDS in the presence and absence of XAD-7 resin are shown in Figure 4.

#### 2.2.2. Humic Substances

**Figure 5:**
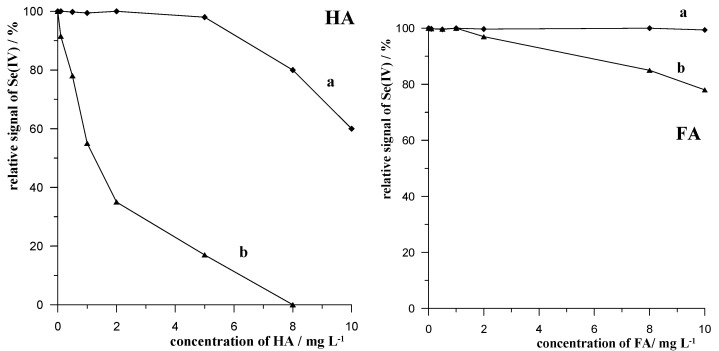

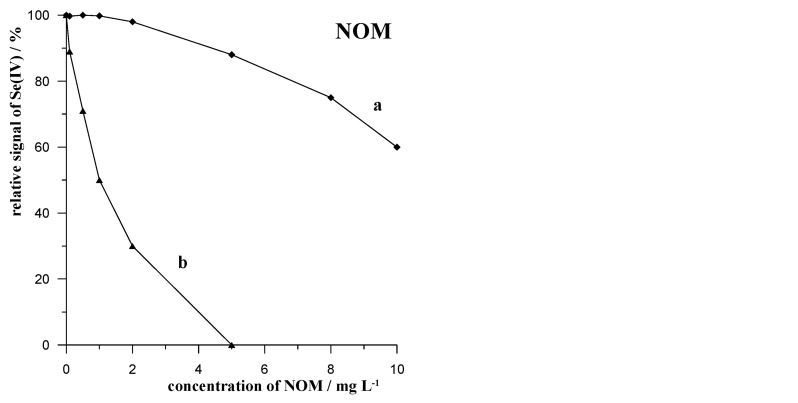
The influence of concentration of HA, FA, and NOM on relative signal of Se(IV) in the absence (a) and presence (b) of the procedure of mixing with resin Amberlite XAD-7.

The effects of the following humic substances, HA, FA, and NOM, on the analytical Se(IV) signal were precisely investigated. The tests were carried out in the same way as those for the surfactants, and the results obtained are presented in Figure 5. For comparison, each graph also shows the Se(IV) signal recorded in the presence of each humic substance, but without the presence of the Amberlite XAD-7 resin. As can be seen, in each case, the presence of the resin reduced the interference caused by the humic substances, but the effect was not as spectacular as that in the case of the surfactants. It should also be noted that humic substances attenuated the Se(IV) signal, but to a much lesser extent than the surfactants. However, the use of mixing with resin at 50 °C was shown to produce an undisturbed Se(IV) signal in the presence of 5 mg L^−1^ of HA, 10 mg L^−1^ of FA, and 2 mg L^−1^ of NOM.

### 2.3. Analysis of Environmental Water Samples

The developed sample preparation procedure prior to voltammetric Se(IV) analysis using an environmentally friendly solid bismuth microelectrode was used to analyse the certified reference materials SPS-SW1 (surface waters, Spectrapure Standards, Oslo, Norway) and TM-25.5 (Lake Ontario water, Environment and Climate Change, Canada). In addition, the materials were enriched with different concentrations of surfactants and humic substances. Due to the strongly acidic environment of the materials analysed, an additional introduction of NaOH was necessary to achieve the desired pH. The results obtained are presented in Table 1. As can be seen, satisfactory results were obtained in each case, confirming the effectiveness of the proposed procedure for preparing actual samples prior to measurement.

## 3. Materials and Methods

### 3.1. Reagents

Acetate buffers were prepared from acetic acid and sodium hydroxide (Suprapur, Merck, Rahway, NJ, USA). A stock solution of 1 g L^−1^ of Se(IV) was obtained from Sigma Aldrich (St. Louis, MO, USA). Standard solutions of Se(IV) and other ions were prepared by dissolving appropriate amounts of their nitrate or chloride with deionized water. Triton X-100, sodium dodecyl sulphate (SDS), cetyltrimethylammonium bromide (CTAB), and Rhamnolipid were acquired from Fluka (Buchs, Switzerland). Humic acid sodium salt (HA) was obtained from Aldrich. River fulvic acid (FA) and natural organic material (NOM) were obtained from the Suwannee River and purchased from the International Humic Substances Society (Denver, CO, USA). Amberlite XAD-7, acquired from Sigma (St. Louis, MO, USA), was washed four times in distilled water and dried at a temperature of 50 °C. All solutions were prepared from deionized water produced by a water purification Milli-Q system (Millipore, London, UK) and analytical-grade or Suprapur reagents.

### 3.2. Equipment

The investigations were carried out with a µAutolab (Eco Chemie, Utrecht, The Netherlands) with the GPES 4.9 measurement software package. A three-electrode cell (10 mL) was used with a solid bismuth microelectrode (Ø = 25 µm) as a working electrode, a Ag/AgCl electrode as a reference electrode, and a platinum wire electrode as a counter electrode. A detailed description of the design of the solid bismuth microelectrode is presented in the paper [40]. Every day prior to usage, the working microelectrode was polished on 2500 grit sandpaper, subsequently rinsed thoroughly with copious amounts of deionized water, and kept in an ultrasonic bath for 30 s to dislodge any residual polishing material.

### 3.3. Procedure

In order to select the most optimal conditions for the simultaneous sample preparation and voltammetric measurement taking place in the same vessel, suitable solutions were prepared. Each solution contained a fixed concentration of the basic electrolyte acetate buffer at pH = 4.0 of 0.1 mol L^−1^, a fixed concentration of Se(IV), 0.5 g of Amberlite XAD-7 resin, and various concentrations of surfactants or humic substances. Every sample was placed into the thermostated voltammetric cell, and before voltammetric measurement, it was stirred for 2 min. During this time, sample preparation preceding the measurement took place by removing surfactants and/or humic substances by adsorption onto the resin. The voltammetric measurement then began, first by activating the bismuth microelectrode (BiµE) for 2 s as a result of applying a potential of −2.5 V (during this stage, bismuth oxides that may form on the electrode surface were reduced to a metallic form), followed by selenium accumulation at an electrolysis potential of −0.55 V for 30 s. Next, for the stripping step, after 5 s of the rest period, the BiµE was polarized in the range of potentials from −400 mV to −1000 mV and the DP voltammogram was registered. The voltammetric parameters for the determination of Se(IV) on a solid bismuth microelectrode have been previously optimized and described in the literature [40]. Measurements were carried out at a constant temperature ranging from 20 to 60 °C, as required. Unless otherwise stated, measurements were carried out at room temperature. The intensity of the peak current was directly proportional to the concentration of Se(IV) contained in the solution and was used for quantitative analysis. Under such conditions, the calibration graph for Se(IV) for an accumulation time of 50 s was linear in the range from 4 × 10^−9^ to 3 × 10^−6^ mol L^−1^. Compared to other voltammetric procedures for the determination of Se(IV), this has one of the widest ranges of linearity in very low concentration ranges combined with a short measurement time of 50 s. Very important from the point of view of speciation analysis is the fact that only the Se(IV) present in the sample undergoes an electrode reaction in the procedure described, while Se(VI) is electrode-inactive and does not affect the signal. This makes it possible to determine trace amounts of the most toxic form of selenium, i.e., Se(IV), even in the presence of large excesses of Se(VI).

## 4. Conclusions

The preparation of real samples prior to measurement is crucial for final analysis results. In this work, a simple, low-cost, and fast procedure was proposed that allows for sample preparation prior to voltammetric Se(IV) analysis using an environmentally friendly solid bismuth microelectrode. The results proved that it was possible to analyse trace amounts of Se(IV) in the presence of up to 10 mg L^−1^ of surfactants and surface matter. The negative effect on the analytical selenium signal was found to depend on the surfactant load, as well as the type of humic substance. However, in any case, mixing the sample with resin at 50 °C eliminated or reduced these interferences, and the elimination effect was significantly greater for surfactants than for humic substances. A great advantage of the proposed procedure is that the mixing of the sample with the resin at an elevated temperature is carried out in the same vessel and solution, in which the voltammetric measurement is carried out immediately. The use of XAD-7 resin to remove the organic matrix from environmental samples is in line with green chemistry guidelines and does not involve the use of environmentally harmful reagents necessary to mineralise the sample to remove the organic matrix. The developed procedure was successfully tested by analysing certified reference materials. The matrix of these materials was further enriched with various surfactants and humic substances. In conclusion, it can be stated that thus procedure for the preparation of natural water samples prior to Se(IV) analysis can be widely used in analytical laboratories due to its advantages.

## Figures and Tables

**Table 1:** Results of Se(IV) determination in certified reference materials SPS-SW1 and TM-25.5 enriched in surfactants and humic substances.

Sample	Certified Value ± SD [µg L^−1^]	Type of Interferent Added	Concentration of Added Interferent [mg L^−1^]	Se(IV) Determined ± SD [µg L^−1^]
SPS-SW1 (surface water)	2.00 ± 0.02	-	-	2.12 ± 0.08
		Triton X-100	7	1.88 ± 0.12
		SDS	3	1.85 ± 0.14
		HA	4	2.15 ± 0.10
TM-25.5 (Lake Ontario water)	29.2 ± 3.5	-	-	30.4 ± 2.6
		CTAB	8	30.5 ± 3.1
		Rhamnolipid	4	28.0 ± 2.5
		FA	8	28.8 ± 3.3

## Data Availability

Data are contained within the article.
